# Pseudophakic mini-monovision: high patient satisfaction, reduced spectacle dependence, and low cost

**DOI:** 10.1186/s12886-018-0963-3

**Published:** 2018-11-09

**Authors:** Debora Goetz Goldberg, Michael H. Goldberg, Riddhi Shah, Jane N. Meagher, Haresh Ailani

**Affiliations:** 1 Department of Health Administration and Policy, George Mason University Peterson Family Health Sciences Hall, 4400 University Drive, MS: 1J3, Fairfax, Virginia 22030 USA; 2 Eye Consultants of Northern Virginia, 8134 Old Keene Mill Rd., Ste. 300, Springfield, VA 22152 USA

## Abstract

**Background:**

Cataract surgery with pseudophakic mini-monovision has lower out-of-pocket patient expense than premium multifocal intraocular lenses (IOL). The purpose of this study was to evaluate patient-reported satisfaction and spectacle dependence for key activities of daily living after cataract surgery with pseudophakic mini-monovision. The study also examined statistical relationships between patient demographic variables, visual acuity and satisfaction.

**Methods:**

Prospective cohort study of 56 patients (112 eyes) who underwent bilateral cataract surgery with pseudophakic mini-monovision. Mini-monovision corrects one eye for distance vision and the other eye is focused at near with − 0.75 to − 1.75 D of myopia. All patients with 1 diopter or greater of corneal astigmatism had a monofocal toric IOLs implanted or limbal relaxing incision. The main study outcomes were assessed at the last follow-up appointment and included refraction, visual acuity, patient reported spectacle use, and patient satisfaction. Descriptive statistics, correlation matrixes and Pearson’s chi-square tests were examined.

**Results:**

Uncorrected visual acuity was significantly better post-operatively. Most patients reported the surgery met their expectations for decreased dependence on spectacles (93%). Most patients report little or no use of spectacles post-operatively for computer use (93%), distance viewing (93%) and general use throughout the day (87%). A small number of patients report spectacle use for reading (9%) and night driving (18%). There were no relationships detected between demographic variables and visual acuity or patient satisfaction.

**Conclusions:**

Aging of the population presents one of the biggest challenges in the health sector, which includes a rising number of individuals with chronic vision impairment and increased demand for accessible treatment strategies. Cataract surgery with pseudophakic mini-monovision results in high patient satisfaction and considerable reduction in spectacle dependence. Pseudophakic mini-monovision technique is a low-cost, valuable option for patients who would like to reduce dependence on spectacles post-operatively and should be considered along with premium multifocal IOLs in options available for patients based on their needs, preferences and clinical indicators. Reducing spectacle dependence with the pseudophakic mini-monovision technique could improve the functionality, independence and quality of life for many patients who are unsuitable or are unable to pay additional fees associated with premium multifocal IOLs.

## Background

Decreased spectacle dependence with a minimum of optical disturbances has become a major refractive goal of cataract surgery. However, patients with traditional cataract surgery are often dependent on spectacles post-operatively. Several surgical options exist for reducing spectacle dependence including bilateral diffractive multifocal intraocular lenses (IOLs) and traditional monofocal IOLs with pseudophakic mini-monovision. Multifocal IOLs are increasingly used to reduce spectacle dependence, although these IOLs may not be a feasible option for many patients due to additional out-of-pocket expense patients may incur for facility and physician charges attributable to multifocal IOLs [1].

Monovision using traditional monofocal IOLs is a surgical option that corrects distance vision in the dominant eye; the non-dominant eye focuses intentionally for near to mid-range vision. This leads to the process of neuroadaptation where the brain can use the distance image from the dominant eye and the near image from the non-dominant eye to achieve a wider range of functional vision [2]. In this surgery the amount of intended myopia can vary, with full monovision defined as the reading eye exhibits a residual refractive error of − 2.50 diopters or more. Modified monovision or “mini-monovision” requires a smaller interocular diopteric power difference between eyes than traditional monovision, typical calculations of the near eye are anywhere between −.75 and − 1.75 diopters of myopia. Patients with significant astigmatism can also undergo mini-monovision surgery along with toric IOL implants or limbal relaxing incisions. Previous research has found that IOLs implanted bilaterally using the mini-monovision approach result in few optical side effects and exceptional distance and intermediate visual outcomes. Concerns remain regarding near visual outcomes that require some patients to wear spectacles for reading fine print or computer work [3].

Both multifocal IOLs and monofocal IOLs with mini-monovision technique have been found to significantly improve uncorrected visual acuity and spectacle independence [4–8]. A few studies comparing multifocal IOLs with the mini-monovision technique found similar results between the groups on visual acuity [5, 7] and spectacle independence [6, 9]. However, challenges remain after cataract surgery with multifocal IOLs due to visual aberrations such as halos, glare, shadows, waxy vision, and difficulty reading in dim light [2, 4, 5, 9–11]. These unwanted visual symptoms following multifocal IOL surgery are common; previous research has found between 30 and 65% of patients report visual aberrations [10]. One study found that up to 10% of patients reported that the glare and halos were debilitating, with many of those patients requiring a lens exchange to correct their symptoms [2].

In the United States, there is economic incentive for surgeons to use multifocal IOLs in lieu of mini-monovision with monofocal IOLs because the physician can legally make a surcharge to the patient for the use of a premium multifocal IOL, which is typically an out-of-pocket expense for patients [7]. High patient out-of-pocket expenses for premium multifocal IOLs is common in many counties. Physicians may also be able to charge additional fees for necessary testing prior to cataract surgery for mini-monovision; however, these fees run considerably less than charges for the premium multifocal IOLs.

The National Academy of Medicine promotes research on approaches to reduce vision impairment that are focused on patient-centered care and low-cost treatment strategies [12]. Mini-monovision is a low-cost, effective option for management of presbyopia and has fewer side effects than multifocal IOLs [4, 5]. Patients often are drawn by the financial savings of mini-monovision with traditional IOLs compared with multifocal IOLs. However, few studies have evaluated patient reported satisfaction with spectacle dependence following mini-monovision with traditional IOLs. The main purpose of this study was to determine if mini-monovision cataract surgery fulfilled patients’ expectations and decreased dependence on spectacle correction for distance, midrange and near functions. A secondary objective of the study was to determine if patient characteristics, such as age and gender, are related to visual acuity or patient satisfaction following surgery.

## Methods

This prospective cohort study was a partnership between Eye Consultants of Northern Virginia (ECNV) and George Mason University, College of Health and Human Services. The ECNV and George Mason University Internal Review Board approved the study protocols. Study outcomes include clinical measures of uncorrected visual acuity and patient-reported data obtained through a questionnaire completed by patients at the 3-month post-operative follow up exam. The questionnaire consisted of 6 items measuring spectacle use for near, mid-range and distance functions such as driving at night, using the computer, and reading. The questionnaire also asked patients to rate their overall satisfaction with mini-monovision cataract surgery for reduction of spectacle dependence. Chart reviews were conducted by ECNV ophthalmologists and ophthalmic technicians to extract patient demographic data and measures of visual function.

### Patient sample

The sample was drawn from all patients at ECNV in need of bilateral cataract extraction who chose the mini-monovision cataract surgery option, which included 63 consecutive patients between 2012 and 2015. Patients who experienced major ocular pathology such as corneal dystrophy, degeneration or macular pathology were excluded from the study. There were 56 patients (112 eyes) who fully completed the questionnaire on spectacle use and satisfaction, resulting in an 89% response rate. All patients provided informed consent prior to surgery and before completion of the questionnaire.

### Preoperative examination

All patients had a complete preoperative ophthalmic examination including subjective and objective refraction, biomicroscopy of the anterior and posterior eye segments, intraocular pressure (IOP), macular optical coherence tomography (OCT), automated and manual keratometry, optical biometry, and immersion A-scan.

### Surgical technique

Pseudophakic mini-monovision was chosen for this study because the gradual difference between the two eyes is more tolerable by patients than full monovision. For purposes of this study, the definition of mini-monovision is the near eye calculated between − 1.25 and − 1.50 diopters spherical equivalent. In all patients the dominant eye was corrected for distance vision. Toric IOLs or limbal relaxing incision were used on patients who had corneal astigmatism greater than 1 diopters. Two patients who previously wore monovision contact lenses, the refractive outcome was calculated for − 2.00 diopters. The same experienced surgeon (M.G.) performed all of the surgeries under topical anesthesia using a standard phacoemulsification procedure. All patients were treated with aspheric IOL implants (AcrySof®IQ IOL or the AcrySof® Toric/IQ IOL).

### Postoperative examination

Routine postoperative examinations were performed 1 day, 3 weeks and 3 months (up to 6 months) after surgery. The exam included testing for visual acuity, visual aberrations, IOP, subjective and objective refraction and biomicroscopy of the anterior segment. The main study outcomes were assessed at the last follow-up visit. Outcomes included visual acuity, visual aberrations, patient reported questionnaire data on spectacle usage for common Activities of Daily Living (ADLs), and patient satisfaction with reduction of spectacle dependence following the mini-monovision technique.

### Statistical analysis

Statistical analyses were performed using STATA 14 software. Measures of central tendency and dispersion are provided on patient demographic variables and responses to survey questions. Correlation matrixes and Pearson’s chi-square tests were examined to determine associations between patient demographic variables, visual acuity, and overall patient satisfaction with reduction of spectacle dependence following surgery. The paired-samples t-test was performed to compare pre- and postoperative visual acuity means (converted to logMAR units) to test for statistical significance. A *P* value of less than .05 was considered statistically significant for this study.

## Results

This study included 112 eyes of 56 patients. Cataract surgery was uneventful in all cases and there were no intraoperative complications. All patients completed the questionnaire on spectacle dependence and attended the last follow-up visit. The sample comprised of 39 female and 17 male patients between the ages of 55 and 80.

Patient demographic information and surgical procedures are presented in Table 1. Additional procedures were performed on patients to correct astigmatism. This included 10 eyes treated with limbal relaxing incision. Two patients who had previous refractive surgery underwent IOL exchange on the dominant eye to achieve the desired post-operative refraction.

**Table 1:** Patient Sample Demographics (*n* = 56)

Demographic Variable	% (n)
Participant Age	
55–64	14.3 (8)
65–74	64.3 (36)
75+	21.4 (12)
Gender	
Male	30.4 (17)
Female	69.6 (39)
Additional Procedures	
Limbal Relaxing Incision OD	8.9 (5)
Limbal Relaxing Incision OS	8.9 (5)
IOL Exchange OD	1.8 (1)
IOL Exchange OS	1.8 (1)

### Visual acuity

The preoperative and postoperative visual acuity are reported in Table 2 in logMAR units. The postoperative uncorrected near and distance visual acuities were significantly better than preoperative measurements in the patient sample (*P* < .001). The target refraction for the distance eye was plano and the near eye was − 1.25 to − 1.50. Fifty-one patients (91%) were within ±.50 diopters of the intended spherical equivalent for the distance eye. Fifty-two patients (93%) were within ±.50 diopters of the target range for the near eye. There were no patient reports of meaningful glare or halos for near or distance functions.

**Table 2:** Pre and Post Surgery Visual Acuity Characteristics (n = 56)

Pre-Surgical Measurements	Mean ± SD (logMAR units)
Uncorrected Vision OD	0.95 ± 0.45
Uncorrected Vision OS	0.9 ± 0.44
Post-Surgical Measurements	Mean ± SD (logMAR units)
Uncorrected Vision Distance Eye	0.09 ± 0.09
Uncorrected Vision Near Eye	0.15 ± 0.14

### Patient reported satisfaction and spectacle use

All patients completed a questionnaire on spectacle use and satisfaction, see Table 3. One question asked patients if the mini-monovision technique met their expectations for decreased dependence on spectacles. This satisfaction scale ranged from 1 to 10, with 1 representing the lowest level of satisfaction and 10 representing the highest level of satisfaction. The research findings show most patients reported cataract surgery with mini-monovision technique met their expectations for decreased dependence on spectacles. On this question 51 (93%) patients reported 7 or higher on a 10-point satisfaction scale, with only one (2%) patient reporting a 3 or less on their level of satisfaction.

**Table 3:** Patient Reported Spectacle Use and Satisfaction (*n* = 56)

Patient Survey Question	Mode	Mean	Std Dev
1. Time Wearing Glasses for Reading	0	2.4	2.8
2. Time Wearing Glasses at Computer	0	0.9	2.0
3. Time Wearing Glasses for Distance	0	0.7	1.7
4. Time Wearing Glasses for Driving at Night	0	2.1	3.8
5. Time Wearing Glasses throughout the Day	0	1.6	1.6
6. Mini-Monovision Surgery Met Patient Expectations for Decreased Dependence on Glasses	10	9.3	1.6

Note: Questions 1–5 based on a 0 to 10 scale and question 6 based on a 1 to 10 scale

Questions on spectacle use were based on a frequency scale from 0 to 10, with 0 representing never wearing spectacles and 10 representing always wearing spectacles. Overall, patients reported low use of spectacles for specific activities for distance, mid-range and near functions. Patient report of spectacle use was lowest for computer use, distance viewing, and for general use throughout the day. The majority of patients 51 (93%) reported low scores (0, 1, 2 or 3) for the amount of time they wear spectacles while working on a computer. Likewise, most patients, 51 (93%), also reported low scores (0, 1, 2 or 3) for the amount of time they wear spectacles for distance viewing. Slightly fewer patients, 48 (87%), reported low scores (0, 1, 2 or 3) for the amount of time they wear spectacles throughout the day.

The highest reported use of spectacles was for reading and for driving at night. Most patients, 41 (73%), reported low scores (0, 1, 2, 3) for wearing spectacles for reading, with 5 (9%) patients reported high scores (7, 8, 9, 10). Similarly, 42 patients (76%) reported low scores (0, 1, 2, 3) for wearing spectacles for night driving; however, 11 (18%) patients reported high scores (7, 8, 9, 10) for wearing spectacles for driving at night. The study findings indicate that for most patients cataract surgery with the mini-monovision technique relieved their need for spectacles for distance, mid-range and near functions. Nevertheless, a small number of patients need vision correction with spectacles for reading and driving at night.

### Additional analysis

Correlation matrixes and Pearson’s chi-square tests were examined to determine associations between patient demographic variables, visual acuity and satisfaction. No associations were found between gender and post-operative uncorrected visual acuity in the distance eye (Pearson chi2 2.54, *P* = .467) or near eye (Pearson chi2 3.44, *P* = .486). No association was found between age and post-operative uncorrected visual acuity in the distance eye (Pearson chi2 54.10, *P* = .780). However, a statistically significant positive association was found between age and post-operative uncorrected visual acuity in the near eye (Pearson chi2 117.66, *P* = .009). No associations were found between patient age and patient satisfaction (Pearson chi2 127.24, *P* = 0.452), nor were there associations between patient gender and satisfaction (Pearson chi2 7.69, *P* = 0.262). A statistically significant positive association was found between post-surgery uncorrected visual acuity and patient satisfaction, indicating that patients with better vision were more satisfied with cataract surgery involving the mini-monovision technique (Pearson chi2 30.32, *P* = .034).

## Discussion

Results of this study found that cataract surgery with the mini-monovision technique significantly improved patients’ uncorrected visual acuity and reduced their spectacle dependence. Almost all patients in the study reported that cataract surgery with the mini-monovision technique met their expectations for decreasing dependence on spectacles, with most patients reporting little or no use of spectacles post-operatively. Patients reported low use of spectacles for computer work, distance viewing, and general activities throughout the day. The quality and levels of light and/or the distance from the object may be related to the need for a small number of patients to wear spectacles postoperatively for reading and driving at night. Previous research has found that patients who receive either monofocal or multifocal IOL for cataract surgery have issues with night driving [13]. Other studies found that patients undergoing cataract surgery with multifocal IOLs experience more difficulties with night vision and night driving than patients undergoing cataract surgery with monovision [14].

Study results also found a positive relationship between post-surgery uncorrected visual acuity and patient satisfaction, indicating that patients with better vision were more satisfied with cataract surgery involving the mini-monovision technique. While there was significant improvement in visual acuity postoperatively, patients with poorer outcomes were less satisfied with pseudophakic mini-monovision for spectacle dependence. It is also possible that postoperative astigmatism interferes with near, mid-range and distance visual acuity, and therefore with patient satisfaction and spectacle dependence.

Results of this study, along with other research [15], indicate that cataract surgery with pseudophakic mini-monovision is a successful option to lessen patient dependence on spectacles for near, midrange and distance functions after cataract surgery. Cataract surgery with multifocal IOLs has also been shown to be an effective method for reducing dependence on spectacles. For many patients, multifocal IOLs are an appropriate and valuable service. Nevertheless, many previous studies have shown that some patients experience optical side effects, such as glare, halos, and dysphotopsia, which can cause patient discomfort and decreased satisfaction [10]. The dissatisfaction from multifocal IOLs often cannot be improved with spectacles and instead requires additional surgery such as IOL exchange to a monofocal IOL. In contrast, patient dissatisfaction with the pseudophakic mini-monovision technique is mostly related to the refractive outcome, which can be corrected with spectacles or contact lenses and does not require additional surgery. A systematic review of research on quality outcomes revealed that patients with multifocal IOLs are more likely than patients with pseudophakic mini-monovision technique to undergo IOL exchange due to dissatisfaction with image quality [16].

The decision to opt for either cataract surgery with multifocal IOLs or cataract surgery with the mini-monovision technique should be based on consideration of patient motivation to achieve spectacle independence [11], patient ADLs [15, 17], treatment costs, and ability to cope with possible side effects. An understanding of the benefits and risks of both pseudophakic mini-monovision and multifocal IOLs is critical for patients to be informed decision makers. The move toward patient-centered care supports the need for patient engagement and informed decisions about treatment options. Patient-centered care is “providing care that is respectful of and responsive to individual patient preferences, needs, and values, and ensuring that patient values guide all clinical decisions” [18]. Informed consent, one component of patient-centered care, includes discussion of: the illness and the natural consequences of no treatment; detailed information on the proposed operation, including commonly known complications; and alternative forms of treatment [19]. Physicians should advise their patients on all possible options for reducing dependency on spectacles with cataract surgery including potential side effects and additional out-of-pocket costs. Providing patients with complete information about expected benefits and risks, and expected costs, will lead individuals to be more confident in the recommended choice [20].

Results of this study should be considered along with study limitations, which include a small sample size, limited number of ADLs assessed in the questionnaire, and the lack of comparison data on patient satisfaction outcomes with other options to reduce patient dependence on spectacles following cataract surgery. Future studies should focus on assessment of spectacle dependence when performing ADLs with technologies such as cellular messaging and cellular entry search [17]. Future studies should also consider healthcare practitioner assessment of ADLs following cataract surgery with mini-monovision. Additional Randomized Controlled Trials are needed to compare efficacy of the mini-monovision technique with cataract surgery using multifocal IOLs [21]. There is also a need for research on the role of costs in patient decision making for selecting techniques to reduce dependence on spectacles.

## Conclusion

Aging of the population presents one of the biggest challenges in the health sector, which includes a rising number of individuals with chronic vision impairment and increased demand for accessible treatment strategies. Pseudophakic mini-monovision is a low-cost option for patients who would like to reduce dependence on spectacles post-operatively and should be considered along with multifocal IOLs in options available for patients based on their needs, preferences and clinical indicators. Reducing spectacle dependence with the mini-monovision technique could improve the functionality, independence and quality of life for many patients who are unsuitable for multifocal IOLs or are unable to pay additional fees associated with multifocal IOLs.

## Abbreviations

ADLs: Activities of daily living
ECNV: Eye Consultants of Northern Virginia
IOLs: Intraocular lens
IOP: Intraocular pressure
OCT: Optical coherence tomography
RCT: Randomized Controlled Trials
Std dev: Standard Deviation
